# Cancer datasets and the SARS-CoV-2 pandemic: establishing principles for collaboration

**DOI:** 10.1136/esmoopen-2020-000825

**Published:** 2020-05-25

**Authors:** Carlo Palmieri, Daniel Palmer, Peter JM Openshaw, J Kenneth Baillie, Malcolm G Semple, Lance Turtle

**Affiliations:** 1 Department of Molecular and Clinical Cancer Medicine, Institute of Systems, Molecular and Integrative Biology, University of Liverpool, Liverpool, UK; 2 Department of Medical Oncology, Clatterbridge Cancer Centre NHS Foundation Trust, Liverpool, UK; 3 National Heart and Lung Division, Faculty of Medicine, Imperial College, London, United Kingdom; 4 Roslin Institute, University of Edinburgh, Edinburgh, UK; 5 Intensive Care Unit, Royal Infirmary of Edinburgh, Edinburgh, UK; 6 Alder Hey Children's NHS Foundation Trust, Liverpool, UK; 7 NIHR Health Protection Research Unit in Emerging and Zoonotic Infections, Faculty of Health and Life Sciences, University of Liverpool, Liverpool, United Kingdom; 8 Tropical and Infectious Disease Unit, Liverpool University Hospitals NHS Foundation Trust, Liverpool, UK

**Keywords:** cancer, COVID-19, principles

Dear Editor,

The severe acute respiratory syndrome coronavirus 2 (SARS-CoV-2) pandemic has been a major disruptive event for the global oncology community. It has challenged and compromised the delivery of oncological care as a result of (1) the diversion of resource to support the care of acutely and critically ill patients with COVID-19; (2) a reduction in the number of highly trained staff who deliver such treatments due to sickness, self-isolation or family reasons^1^; and (3) concerns related to treating patients with cancer, given issues related to the potential risks of acquiring SARS-CoV-2 and the degree of severity of COVID-19 as a result of either innate or iatrogenic cancer-related immunodeficiency. The current peer-reviewed data regarding the course of COVID-19 in patients with cancer is limited to retrospective cases series of 11–105 patients with variability in the data reported^2–6^ and one study involving aggregate-level data from 334 patients.^7^ These datasets do not enable the identification of risk factors that might predispose to symptomatic SARS-CoV-2 infection nor do they identify those factors that predict for serious morbidity and mortality as a result of infection. Such information is urgently needed to inform the development of a robust evidence base approach to risk stratification by tumour and treatment type, as well as development and introduction of appropriate mitigation measures.

It is in response to this information vacuum that a number of cancer-specific observational studies and audits have been developed in an organic and parallel manner (table 1). These studies cover surgical, oncological and psychological aspects of COVID-19. They range from the collection of data on all cancers to information on specific cancers; most are retrospective, involving a single specialist group. Some do take a cross specialty approach and enable comparison to non-cancer cohorts, as well as enable translational research from biological samples (table 1). In addition to these, there are audits led by specialist societies, such as Intensive Care National Audit and Research Centre, which provide some information on cancer cases with the potential to be analysed in great detail and to allow comparison with patients without cancer.^8^ The International Severe Acute Respiratory and Emerging Infections Consortium WHO Clinical Characterisation Protocol has collected detailed clinical information and outcomes for over 30 000 people of all ages admitted to hospitals with COVID-19 and has recorded major comorbidities and concomitant medications that identify those people affected by cancer.^9^

**Table 1:** Summary of the current cancer observational and translational studies related to the SARS-CoV-2/COVID-19 pandemic

Name of study/location	Brief description
CovidSurg–Cancer/global	Observational Evaluate the 30-day COVID-19 infection rates in elective cancer surgery during the COVID-19 pandemic (https://globalsurg.org/cancercovidsurg/)
The COVID-19 and Cancer Consortium The USA, the European Union, Argentina, Canada and the UK are eligible to participate. Currently, there are 100 USA centres.	Observational Aim is to collect data about patients with cancer who have been infected with COVID-19 (https://ccc19.org/)
American Society of Haematology Research Collaborative COVID-19 Registry for Hematologic Malignancy/global	Observational Captures data on people who test positive for COVID-19 and have been or are currently being treated for hematological malignancy (https://www.ashresearchcollaborative.org/covid-19-registry)
Thoracic Cancers International COVID-19 Collaboration/global	Observational A global consortium designed to gather information on patients with thoracic cancer infected with COVID-19 regardless of therapies administered (http://www.etop-eu.org/index.php?option=com_content&view=article&id=115644&catid=13&Itemid=557)
Clinical Characterisation Protocol–Cancer UK/UK	Prospective observational and biological samples The study will characterise the presentation, management and outcome of patients with solid and haematological malignancies recruited into the prospective Clinical Characterisation Protocol for Severe Emerging Infections in the UK. It will also compare patients with cancer to those without cancer. The biology of SARS-CoV-2 in the context of cancer-associated or iatrogenic immunosuppression will also be investigated (https://isaric.tghn.org/UK-CCP/)
UK Coronavirus Cancer Monitoring Project/UK	Observational The UK Coronavirus Cancer Monitoring scheme is a clinician-led reporting project recoding data related to patients with cancer who have tested positive for COVID-19 across the UK (https://ukcoronaviruscancermonitoring.com/). Paediatrics (https://ukcoronaviruscancermonitoring.com/paediatrics/)
ONCOVID/UK, Italy and Spain	Observational To describe the features of COVID-19 infection in patients with cancer, investigate its severity in this population and evaluate long-term outcomes (https://www.oncovid.net/)
UK COVID and Gynaecological Cancer Study/UK	Observational Records and assesses changes and outcomes in patients across the whole patient pathway and within the multidisciplinary team context bb-ukcogs@qmul.ac.uk
Patients with AML and COVID-19 Epidemiology/UK	Observational Aims to understand the incidence, presentation and severity of COVID-19 during treatment of AML As well as to develop informed recommendations for the care of patients with AML, including those who develop COVID-19 infection during treatment or have recovered from prior COVID-19 infection
COVID-RT Clinicaland Translational Radiotherapy (CT-RAD) Research Working Group, UK	Observational Aimsto capture changes in radiotherapy pathways and understand their impact onradiotherapy services and patient outcomes across the UK. The initiative willnot only focus on patients with COVID-19, but all radiotherapy patients (https://www.ncri.org.uk/news/covid19-radiotherapy-initiative/)
The American Society of Clinical Oncology Survey on COVID-19 in Oncology Registry/USA	Observational Captures baseline and follow-up data on how the impact of SARS-CoV-2 on cancer care and cancer patient outcomes during the COVID-19 pandemic and into 2021
Psychology study/China	Observational The effects of prevention and control measures on treatment and psychological status of patients with cancer during the COVID-19 outbreak (http://www.chictr.org.cn/showproj.aspx?proj=50714)
Clinically related study/China	Observational/retrospective Clinical characteristics and prognosis of patients with cancer with COVID-19 based on bioinformatics analysis (http://www.chictr.org.cn/showproj.aspx?proj=51019)
Perioperative immune prediction and intervention of patients with tumour undergoing surgery during the COVID-19 outbreak period/China	Interventional/prospective To understand the influence of the pandemic on the prognosis of patients undergoing cancer surgery and to understand the influence of different interventions on outcomes (http://www.chictr.org.cn/showproj.aspx?proj=50984).

AML, acute myeloid leukemia; SARS-CoV-2, severe acute respiratory syndrome coronavirus 2.

It is key that all these important efforts culminate in a robust evidence which can enable^1^ governments and policy makers to provide clear advice regarding the need or otherwise for patients with cancer to self-isolate/cocoon, as well as to identify groups to prioritise for vaccination or other evidence-based interventions which might reduce the severity of infection^2^; oncologists to provide clear advice regarding the risks of specific treatment modalities and systemic anticancer therapy for specific cancers in the era of SARS-CoV-2 and^3^ patients to make more informed decisions regarding their cancer care and the degree they choose interact and mix at societal and family levels. The latter is particularly important for patients with life-limiting diagnoses, as well as for addressing the mental health effects of self-isolation.^10^

To enable this and to ensure we harness the true potential of all these data, we wish to suggest the adoption of what we have named ‘principles for collaboration in the field of cancer and COVID-19’. These principles are (1) the establishment of a searchable database of all non-IMP (Investigational medicinal products) COVID-19 cancer studies with all protocols and documents being made available; (2) enabling patients with cancer to register and contribute their own data and biological material if they so wish; (3) establishment of an agreed core cancer COVID-19 dataset with accepted common definitions, such as defining events and severity of infection; (4) the involvement of experts in infectious disease, microbiology, infection control and critical care in all projects, given the cross-cutting nature of COVID-19 and the need to capture relevant data across theses specialities; (5) agreement to bring all datasets together for a meta-analysis; and (6) the creation of a public facing open-access repository of all data for future research and policymaking. We hope that the oncology–COVID research community that has developed since the inception of the pandemic can cooperate and coordinate using these principles for the benefit of our patients and society.
